# Precision Oncology in Metastatic Uterine Cancer; Croatian First-Year Experience of the Comprehensive Genomic Profiling in Everyday Clinical Practice

**DOI:** 10.3389/pore.2021.1609963

**Published:** 2021-09-27

**Authors:** Dora Čerina, Višnja Matković, Kristina Katić, Ingrid Belac Lovasić, Robert Šeparović, Ivana Canjko, Blanka Jakšić, Ana Fröbe, Stjepko Pleština, Žarko Bajić, Eduard Vrdoljak

**Affiliations:** ^1^ Department of Oncology, School of Medicine, University Hospital Center Split, University of Split, Split, Croatia; ^2^ Department of Gynecologic Oncology, University Hospital Center Zagreb, Zagreb, Croatia; ^3^ Department of Radiotherapy and Oncology, University Hospital Center Rijeka, Rijeka, Croatia; ^4^ Department of Medical Oncology, Division of Medical Oncology, University Hospital for Tumors, Sestre Milosrdnice University Hospital Center, Zagreb, Croatia; ^5^ Department of Radiotherapy Oncology, University Hospital Center Osijek, Osijek, Croatia; ^6^ Department of Oncology and Nuclear Medicine, Sestre Milosrdnice University Hospital Center, Zagreb, Croatia; ^7^ Department of Oncology, University Hospital Center Zagreb, Zagreb, Croatia; ^8^ Research Unit “Dr. Mirko Grmek”, University Psychiatric Hospital “Sveti Ivan”, Zagreb, Croatia

**Keywords:** targeted therapy, genomic profiling, uterine cancer, precision oncology, mutation

## Abstract

Comprehensive genomic profiling (CGP) is gradually becoming an inevitable part of the everyday oncology clinical practice. The interpretation and optimal implementation of the results is one of the hot topics of modern-day oncology. According to the recent findings, uterine cancer harbors a high level of gene alterations but is still insufficiently explored. The primary goal of this project was to assess the proportion of patients with targetable mutations. Also, the aim was to define and emphasize potential opportunities as well as the problems we have faced in the first year of testing on the national level. We performed a multicentric, retrospective, nested cross-sectional analysis on the total population of Croatian patients with advanced/metastatic uterine cancer where the tumor CGP was performed during 2020. CGP of the tumor tissue of 32 patients revealed clinically relevant genomic alterations (CRGA) in 27 patients (84%) with a median of 3 (IQR 1-4) CRGA per patient. The most common CRGAs were those of phosphatide-inositol-3 kinases (PIK3) in 22 patients (69%), with 13/22 (59%) of those patients harboring PIK3CA mutation. The next most common CGRAs were ARID1A and PTEN mutations in 13 (41%) and 11 (34%) patients, respectively. Microsatellite status was determined as stable in 21 patients (66%) and highly unstable in 10 patients (31%). A high tumor mutational burden (≥10Muts/Mb) was reported in 12 patients (38%). CGP analysis reported some kind of targeted therapy for 28 patients (88%). CGP determined clinically relevant genomic alterations in the significant majority of patients with metastatic uterine cancer, defining it as a rich ground for further positioning and development of precision oncology.

## Introduction

Revolutionary advancement of diagnostics through the optimal implementation of informational technologies and development of bioinformatics, combined with a better understanding of the human genome and discovery of the comprehensive genomic testing, has led towards a more individualized and targeted approach to the patient, making the first half of the 21st century a paradigm shift in the establishment of postulates of precision medicine. Consequently, dramatic changes are about to happen when approaching the patient, with taking into consideration his/her known gene alterations when choosing the treatment and their impact on response to it, comorbidities, general condition, as well as other aspects of an individual such as the lifestyle and environmental factors, and altogether with the aim to create optimal treatment strategy for every patient individually. Oncology, as one of the most propulsive branches of medicine, represents the most fruitful ground for the implementation of precision medicine in everyday clinical practice. Definition of underlying causes of carcinogenesis and progress in the field of molecular biology has enabled the development of novel treatment approaches such as molecular-targeted therapy and immunotherapy with improved outcomes and impact on patient`s survival. For instance, molecular-targeted therapy is already a gold standard for first-line treatment in advanced or metastatic non-small cell lung cancer (NSCLC) [1], melanoma [2], gastrointestinal stromal tumor (GIST) [3], or as maintenance therapy in recurrent ovarian cancer [4]. On the other side, immunotherapy with checkpoint inhibitors is becoming the standard of care for many cancer types, such as skin [5], lung [6], renal [7], or bladder cancer [8], and immunotherapy against specific antigens is standardized as a treatment for early or metastatic HER-2 positive breast cancer [9, 10], metastatic colorectal [11], gastric [12], ovarian [13], or cervical [14] cancer. Despite the above, a conservative systemic approach is still the only treatment option for some human malignancies, including uterine cancer, which, alongside cervical cancer, remains the only entity with worsened overall survival in the USA over the last 20 years [15]. Uterine cancer ranks first in incidence among invasive tumors of the female reproductive system in the developed countries due to its association with older age, better socio-economic status, and unopposed estrogen activity [16]. Unfortunately, 15–20% of patients present with or progress to metastatic disease with a 5-years survival rate of 16% [17]. As previously mentioned, the main treatment strategy for metastatic uterine cancer is chemotherapy or hormonal therapy with fewer than 12 months of the median overall survival [18]. According to the TCGA (The Cancer Genome Atlas) project in 2013, uterine cancer/endometrial cancer is divided into four subgroups based on the genomic profiling of 373 endometrial cancer specimens [POLE ultra-mutated, microsatellite instability group, copy number low (CNL), and copy number high (CNH) groups][19]. The POLE ultra-mutated group, which consisted of 7% of tumors, and the microsatellite instability group of tumors (28% of tumors) are candidates for immunotherapy due to the high neoantigen load and consecutively optimal tumor microenvironment for enhanced cytotoxic T-cell response [19]. Improvement in outcomes of the CNL group (39% of tumors) may be in combination with hormonal therapy and the PI3K/AKT/mTOR pathway inhibitor and for the CNH serous-like group (26% of tumors) treatment with cell cycle regulators and the PI3K/AKT/mTOR pathway inhibitors [19]. At the end of 2019, comprehensive genomic profiling (CGP) provided by Foundation Medicine Inc. (FMI) became free in Croatia [20].

Uterine cancer harbors a high level of gene alterations but is still insufficiently explored. It is ranked fourth in cancer incidence in Croatia with 778 women being diagnosed annually and having a mortality-to-incidence ratio of 0.26 [21], and we thus present first-year CGP data on a country level for patients with newly diagnosed metastatic uterine cancer or whose initial disease had progressed during 2020. The primary goal of this project was to assess a share of patients with opted targetable mutations, while the secondary goal was an assessment of the proportion of patients who have started with the CGP-guided therapy. Also, by defining and emphasizing potential opportunities as well as the problems we are facing in the precision oncology development and implementation of this specific field, the aim was to affirm the CGP of patients with metastatic uterine cancer in everyday clinical practice.

## Methods

### Project Design

We performed a multicentric, retrospective, nested cross-sectional analysis on the total population of Croatian patients who were either newly diagnosed with metastatic uterine cancer or whose initial disease has progressed from January 1 to December 31, 2020, and on whose tumors CGP was performed. This analysis was nested within the baseline measurement of the cohort study aimed to assess the real-world utility of CGP, a next-generation sequencing approach that detects novel and known variants of the four main classes of genomic alterations and genomic signatures in order to provide prognostic, diagnostic, and predictive insights that inform research or treatment decisions for individual patients across all cancer types. The obtained tumor specimen was sampled from the surgery or biopsy of the primary disease or metastases and the formalin-fixed, paraffin-embedded tissue for the analysis was sent as a block and one hematoxylin- and eosin-stained slide or 10 unstained slides with one hematoxylin- and eosin-stained slide. The minimal surface area was 25 mm^2^, and the minimal tumor content was 20%; the optimal was 30% of tumor nuclei, defined as the number of tumor cells divided by the total number of all cells with nuclei. In the case of additional immunohistochemistry for PD-L1, four supplementary unstained slides were requested. The majority of CGP analysis was done through FoundationOneCDx, and FoundationOneHeme was performed only for one patient with sarcoma, and it was carried out in a Clinical Laboratory Improvement Amendments certified, College of American Pathologists accredited laboratory (Foundation Medicine Inc., Cambridge, MA, USA). Once the DNA was extracted, 50–1,000 ng underwent whole-genome shotgun library construction and hybridization-based capture in order to detect alterations of 324 genes in total, of which there were 304 exons related to tumors, one promoter region, one non-coding RNA, and certain regions of introns in 34 frequently rearranged genes in tumors, as well as determination of genomic signatures, such as tumor mutational burden (TMB) and microsatellite status. Illumina^®^ HiSeq 4,000 was used to sequence hybrid capture-selected libraries to a high uniform depth. The typical median depth of coverage was >500x with >99% of exons at coverage >100x. The sequenced regions were analyzed for four different types of alterations-base substitution, deletion or insertion, copy number variation, and gene redistribution in a group of genes associated with the tumor development. The microsatellite status was based on genome-wide analysis of 95 microsatellite loci, while TMB was determined by counting all synonymous and non-synonymous variants present at 5% allele frequency or greater, and the total number was presented as mutations per megabase (Muts/Mb) unit [22, 23, 24]. Depending on the results, patients were potentially administered CGP-guided therapy after progression to or unacceptable toxicity of the standard of care first-line or second-line systemic therapy and without having any approved or reimbursed therapy options for the treatment in accordance with the multidisciplinary team’s decision. If patients were administered with CGP-guided treatment, the records of the course of the treatment were collected alongside the occurrence of side effects and the patient’s overall response. Also, there was radiological evaluation at the 2-months intervals to assess the effects of the targeted therapy and to make a decision on its continuation or termination.

This analysis of real-world data was conducted in six Croatian institutions: the University Hospital Centre Split, University Hospital Center Zagreb, Sestre Milosrdnice University Hospital Centre in Zagreb and their Clinic for Tumors, and the University Hospital Centers in Rijeka and Osijek. The project was approved by Ethics Committees of all participating institutions. Informed consent was obtained from all patients before the data collection. Moreover, all patients signed the informed consent for the CGP analysis via FMI. The data file was anonymized before the analysis and the project was performed in accordance with the World Medical Association Declaration of Helsinki of 1975 as revised in 2013 [25].

### Participants

The targeted population was patients initially diagnosed with metastatic uterine cancer or whose disease has progressed from initially diagnosed local or locoregional disease and on whose tumors CGP was performed in 2020. We planned to include the entire population of patients with metastatic uterine cancer who fulfilled the CGP criteria defined by the Croatian Oncology Society: sufficient tissue for the CGP, good general health (ECOG performance status ≤2), and at least 12 months of life expectancy [20]. Hence, we did not perform the power analysis before the project start. Patients were administered with the first- or second-line standard of care treatment for metastatic uterine cancer: chemotherapy or hormonal therapy, depending on their general condition, other comorbidities, and the physician`s choice. CGP-guided therapy was potentially administered after progression to or unacceptable toxicity of the standard of care first- or second-line systemic therapy and without having any approved or reimbursed therapy options for the treatment and in accordance with the multidisciplinary team decisions.

### Endpoints

The primary endpoint was the proportion of patients having clinically relevant genomic alterations, defined as those with approved targeted therapy in the patient’s tumor type or approved in another tumor type, or with existing clinical trials available. The secondary endpoint was the proportion of patients having targetable mutations receiving designated therapy.

### Statistical Analysis

We described the data by percentages, medians, and interquartile ranges (IQR) using StataCorp 2019 (Stata Statistical Software: Release 16. College Station, TX: StataCorp LLC).

## Results

### Description of Patients and Previous Therapy

In 2020, a total of 32 patients with metastatic uterine cancer were presented to multidisciplinary teams, and CGP was performed on their tumor tissue specimens. The median age was 65 (IQR 59-68) years with a total range from 44 to 79 years (Table 1). The majority of patients, 25 (78%) were in good general condition with an ECOG performance status 0. The most common histological subtype was endometrial adenocarcinoma, which was found in 16 patients (50%). All patients received either chemotherapy or hormonal therapy as standard treatment for metastatic uterine cancer. The median number of prior lines of therapy for metastatic disease was 2 (IQR 1-3) (Table 1). The most common chemotherapy protocol used as first-line treatment was a combination of paclitaxel and carboplatin, while hormonal therapy was comprised of megestrol acetate in the first-line setting and then aromatase inhibitor afterward.

**TABLE 1:** Patients characteristics, disease status, and therapy received prior to comprehensive genomic profiling.

	All patients (*n* = 32)	
	n	(%)
Age at diagnosis of metastatic disease, median (IQR)	65	(59–68)
Year of initial diagnosis
2010–2017	8	(25)
2018	6	(19)
2019	10	(31)
2020	8	(25)
Year of metastatic disease
2017–2018	2	(6)
2019	12	(39)
2020	17	(55)
Metastatic disease at initial diagnosis	10	(31)
Time from initial diagnosis to metastatic disease (months), median (IQR)	8	(2–19)
FIGO classification stage at diagnosis
I	10	(31)
II	4	(13)
III	8	(25)
IV	10	(31)
Histological subtypes
endometrial carcinoma
grade 1	5	(16)
grade 2	9	(28)
grade 3	2	(6)
serous adenocarcinoma	6	(19)
clear cell carcinoma	1	(3)
mixed types
endometrial + serious adenocarcinoma	1	(3)
endometrial + clear cell carcinoma	1	(3)
uterine sarcoma	2	(6)
leimyosarcoma	3	(9)
carcinosarcoma	2	(6)^a^
Number of previous treatment lines for metastatic disease
1	12	(39)
2	11	(35)
3	6	(19)
4	2	6)
ECOG performance status before CGP
0	25	(78)
1	6	(19)
2	1	(3)

Data are presented as number (percentage) of patients if not stated otherwise.

CGP, comprehensive genomic profiling; IQR, interquartile range.

Data were missing for date of metastatic disease and number of previous treatment lines for metastatic disease in 1 (3%) patient.

a Total is <100% due to a rounding error.

### Comprehensive Genomic Profiling

Through CGP we found at least one genomic alteration (GA) in 31 (97%) specimens. Clinically relevant genomic alterations (CRGA) were detected in 27 patients (84%) with a median of 3 (IQR 1-4) CRGA per patient (Table 2). The most common CRGAs reported were those of phosphatide-inositol-3 kinases (PIK3) in 22 patients (69%), with 13/22 (59%) of those patients harboring PIK3CA mutation. The next most common CGRAs were ARID1A and PTEN mutations in 13 (41%) and 11 (34%) patients, respectively (Table 2). In total, 30 patients (94%) had genomic alterations without clinical significance with a median of 3 (IQR 1-5) GA per patient. The most common GA without clinical significance was TP53 mutation, reported in 15 patients (47%). Microsatellite status was determined as stable in 21 patients (66%) and determined as highly unstable in 10 patients (31%). The median tumor mutational burden (TMB) was five (IQR 2-18) mutations per megabase (Muts/Mb) with the total range from 0 to 40. High TMB (≥10Muts/Mb) was reported in 12 patients (38%). After analysis of all CGP reports and all detected GA, some kind of targeted therapy was reported for 28 patients (88%), while there was no reportable therapeutic option for 4 patients (13%). Targeted therapy approved for the patient’s tumor type (on-label therapy) was reported in 1 patient (3%), while targeted therapy approved in other tumor type based on patient’s GA (off-label therapy) was reported in 26 patients (81%). Furthermore, targeted therapy without approval but also driven by patient’s GA was reported in 24 patients (75%). The vast majority of alteration-driven therapies encompassed those included in DNA repairs such as PARP inhibitors, PI3-K/mTOR (phosphoinositide-3 kinase/mammalian target of rapamycin) and Ras/Raf/MEK (mitogen-activated protein kinase) inhibitors, or immune checkpoint inhibitors (Table 3). The most common targeted therapies opted were mTOR inhibitors and immune checkpoint inhibitors. Four patients (12.5%) who have had disease progression on the given standard therapy and without further therapeutically valid options, the CGP-guided targeted therapy was opted based upon the MDT decision and compassionate use program availability.

**TABLE 2:** The results of the comprehensive genomic profiling.

	All patients (*n* = 32)	
	n	(%)
Time from the metastatic disease to the	6	(2–14)
CGP (months), median (IKR)		
Genomic alterations		
any genomic alteration	31	(97)
clinically relevant	27	(84)
clinically not relevant	30	(94)
Number of genomic alterations, median (IQR)		
total number	6	(4–7)
clinically relevant	3	(1–4)
clinically not relevant	3	(1–5)
Number of clinically relevant genomic alterations		
0	5	(16)
1	5	(16)
2–3	10	(31)
4–5	8	(25)
6–13	4	(13)^a^
Clinically relevant genomic alterations		
PIK3 pathway	22	(69)
ARID1A	13	(41)
PTEN	11	(34)
KRAS	5	(16)
PALB	4	(13)
Each of BRCA2, CTNNB1	3	(9)
ERBB2, NRAS, RNF43		
Each of AKT1, FBXW7, FGFR2, PTCH1	2	(6)
Each of ALK, ATM, C378R, C83fs*16	1	(3)
CCND1, CDK-4, FANCL, FGFR1, G132V		
KEAP1, MDM, MDM2, MET, MTOR, NF1		
NF2, PIK3CB, Q1835*, Q546K, R93Q		
RAD54L, STK11		
Microsatellite status		
stable	21	(66)
high instability	10	(31)
not determined	1	(3)
Tumor mutational burden (TMB), median (IQR)	5	(2–18)
Tumor mutational burden (TMB)		
not high	19	(59)
high (≥10 mutations/Mb)	12	(38)
not determined	1	(3)

Data are presented as number (percentage) of patients if not stated otherwise

CGP, comprehensive genomic profiling; IQR, interquartile range.

a Total is >100% due to a rounding error.

**TABLE 3:** Suggested treatment options based on the comprehensive genomic profiling.

Suggested treatment	Targeted biomarker	Prevalence	
		n	(%)
Immune check-point inhibitors (nivolumab, avelumab, atezolizumab, pembrolizumab, durvalumab, cemiplimab)	MSI-high	10	(31)
	TMB>10	12	(38)
mTOR inhibitors (everolimus, temsirolimus)	PIK3-kinases	22	(69)
	AKT1	2	(6)
	NF2	1	(3)
	PTEN	11	(34)
	CTNNB1	3	(9)
	FBXW7	2	(6)
	STK11	1	(3)
PIK3 inhibitor (alpelisib)	PIK3CA	13	(41)
PARP inhibitors	PALB2	4	(13)
(olaparib, niraparib, talazoparib, rucaparib)	BRCA2	3	(9)
MEK inhibitors	NRAS	3	(9)
(binimetinib, cobimetinib, trametinib)	NF1	1	(3)
EGFR/HER2 TKIs (afatinib, lapatinib, dacomitinib, neratinib)	ERBB2	3	(9)
antiHER-2 monoclonal antibodies (trastuzumab, pertuzumab, trastuzumabemtansine)	ERBB2	3	(9)
Hedgehog pathway inhibitors (sonidegib, vismodegib)	PTCH1	2	(6)
TKI (pazopanib)	FGFR2	2	(6)
CDK 4/6 inhibitors (palbociclib, ribociclib)	CDK4	1	(3)

## Discussion

Results from the CGP analysis in our project have shown that the vast majority of patients with metastatic uterine cancer harbors at least one genomic alteration, out of which a significant proportion was clinically relevant. In contrast to the conventional testing, which, by single-target assays, discovers potentially one actionable gene alteration, comprehensive genomic profiling (CGP), by next-generation sequencing, gives detailed insight into tumor gene specifics and brings a new dimension to the treatment options of every cancer patient, thus evolving personalized and precision medicine. Consequently, CGP is gradually being integrated into the diagnostic workup of the different tumor types as a backbone diagnostic tool. However, questions that have arisen with CGP like cost, utility, and clinical benefit, and patient’s and societal expectations were some of the hot topics during recent years [26–29]. As previously mentioned, molecular-targeted therapy is already established as a standard treatment in many tumor types, while its use and value outside of current indications are still under investigation. Clinical studies such as the MOSCATO trial [30] have shown improved outcomes but only in the minority of “hard-to-treat” patients, while the phase 2 SHIVA trial discourages the use of “off-label” molecular-targeted therapy due to unimproved progression-free survival comparing it to the conventional treatment [31]. However, the SHIVA trial was criticized for potential biases due to its design as well as targeted therapy that was administered either as monotherapy in patients with several molecular alterations or was incorrectly matched for some patients [32]. On the other hand, several studies have shown favorable effects of the use of “off-label” molecular-targeted therapy with improved and almost doubled response rates and progression-free survivals [33–37]. Meanwhile, the number of in-human studies regarding the dose-escalation of targeted therapy, for instance, phosphatidylinositol 3-kinase α-selective inhibitor alpelisib in patients with specific mutation such as PIK3CA, has lately been increasing rapidly [38]. In addition, new diagnostic approaches lead towards the discovery of tumor genomic signatures such as microsatellite instability and TMB, and these are so-called “tumor agnostic” biomarkers for which the FDA (Food and Drug Administration) approved immunotherapy regardless of cancer type. Despite the abovementioned turmoil about the cost of CGP, it is strongly encouraged, especially in low-income countries, to perform this if CGP is not available, which involves less expensive but equally informative tests, such as immunohistochemistry staining for mismatch repair status (MMR protein staining) [39]. In oncology today, we have more diagnostic capabilities than ever (like CGP), more and more precise drugs, and, contradictorily, less and less valid evidence for their use in individual patients. Furthermore, with an expected, even more precise, granular approach to the single patient and her/his tumor we would most probably end up with a situation where classical clinical trials would not be able to address the needs of further development of oncology science. Consequently, real-world data and learning from every patient experience and every tumor specificity are about to become the backbone of future research in the field of precision oncology in all tumor types together and especially in subtypes driven by targetable biomarkers.

Our results have shown a high mutation load of uterine cancer with at least one genomic alteration found in almost every patient tested, which is in accordance with the previous observations [40, 41]. Furthermore, a vast majority of patients (84%) have clinically relevant genomic alterations, and the most common were PIK3CA, ARID1A, and PTEN, which is similar to the existing findings of 93% [40] and 91% [41]of CRGAs as well as the prevalence of the alterations. Both studies have shown potential clinical benefit from the administered CGP-guided therapy. However, a study by Rodriguez-Rodriguez et al. observed targeted therapy in ovarian and uterine cancer, with only 25 patients with uterine cancer of all stages included in the study. Also, only nine patients were treated in accordance to the CGP with observed stable disease in two patients and partial response in four patients, but the treatment regimen was not stated [40]. On the contrary, a study by Prendergast et al. included 74 patients with recurrent endometrial cancer with a median age of patients of 61 years and a median number of two prior chemotherapy lines (range 1–4). The results of their study showed a median number of CRGAs of 3 (range 0–7), MSI-high status reported in 18% of patients, and a median TMB of 24.3 (range 11.2–48) Muts/Mb per patient. Also, 24/74 (32%) patients have received a matched therapy according to the CGP results which consisted in the majority of patients of agents targeting PI3K/PTEN/mTOR pathway and immunotherapy (pembrolizumab). Objective responses were seen in 25% of patients, nine patients achieved stable disease with a median duration of treatment of 14.6 months, and two out of six patients treated with immunotherapy have shown partial response, while others had stable disease and the median duration of the treatment was 17 months [41]. Although the study has several limitations, such as a small number of patients, comprehensive genomic profiling on archival specimens, lack of consideration for tumor heterogeneity or possible changes of the molecular subtype of the recurrent endometrial cancer, and only a third of patients receiving targeted therapy, it is the first study that links CGP with clinical benefit in the patients diagnosed with recurrent endometrial cancer and suggests its potential benefit in the routine everyday clinical practice [41].

Our cross-sectional data of all tested patients on the country level have shown similarities with the results in the aforementioned studies. However, being the pilot year of testing, there was only a small number of patients in general and particularly those receiving targeted therapy without enough time-length to assess its impact on the response or survival outcomes. Furthermore, there is a discrepancy in the number of patients tested in each institution, defining the learning curve in the new technology adaptation and potentially different approaches to the value of CGP and its clinical use today. Moreover, different penetration of CGP in everyday clinical practice could be due to the different patient distribution, places of surgery, availability of archived or fresh tissues, and organizational issues. Despite the above-mentioned limitations, the majority of positive results speak in favor of our primary goal and have shown the utility of CGP in everyday clinical practice of patients diagnosed with metastatic uterine cancer. Also, our results show good compliance with the established protocol and adherence to the inclusion criteria for the comprehensive genomic profiling on the country level. The number of treated patients with uterine carcinoma in our analysis is rather small, showing the same problem seen in other studies: lack of organization and a structured approach to the CGP driven therapy. Namely, with existing health insurance setups in the majority of countries and the level of partnership between governmental administration and the pharma industry, it is difficult to foresee a faster and better implementation of treatment in terms of precision oncology development. We need more partnership as well as absolute monitoring and information about the performance of the given therapies according to the CGP at the single patient level. Considering these as the nested cross-sectional data, results of the treatment of our patients will be prospectively monitored over the next 2 years, and the outcomes of the precision oncology approach in metastatic uterine cancer therapy will be carefully analyzed in the future.

In conclusion, our country-based real-world data of the comprehensive genomic profiling of patients with metastatic uterine cancer, despite its limitations, represent a significant resource for the estimation of the value of CGP and personalized therapy based on its findings in everyday oncological practice and are important for further positioning and development of precision medicine in patients with uterine cancer.

## Data Availability

The datasets presented in this study can be found in online repositories. The names of the repository/repositories and accession number(s) can be found in the article/Supplementary Material.
