# Supplementary material for: Precision Oncology in Metastatic Uterine Cancer; Croatian First-Year Experience of the Comprehensive Genomic Profiling in Everyday Clinical Practice
Source: Pathol Oncol Res. 2021 Sep 27;27:1609963. doi: 10.3389/pore.2021.1609963 (PMC8504363; doi:10.3389/pore.2021.1609963)
Supplement: Supplementary file 3 [file DataSheet1.PDF]

FOUNDATIONONE® CDx

Technical Specifications

Intended Use

FoundationOne®CDx (F1CDx) is a qualitative next generation sequencing based *in vitro* diagnostic test that uses targeted high throughput hybridization-based capture technology for detection of substitutions, insertion and deletion alterations (indels), and copy number alterations (CNAs) in 324 genes and select gene rearrangements, as well as genomic signatures including microsatellite instability (MSI) and tumor mutational burden (TMB) using DNA isolated from formalin-fixed paraffin embedded (FFPE) tumor tissue specimens. The test is intended as a companion diagnostic to identify patients who may benefit from treatment with the targeted therapies listed in Table 1 in accordance with the approved therapeutic product labeling. Additionally, F1CDx is intended to provide tumor mutation profiling to be used by qualified health care professionals in accordance with professional guidelines in oncology for patients with solid malignant neoplasms. The F1CDx assay is performed at Foundation Medicine, Inc. sites located in Cambridge, MA and Morrisville, NC.

Table 1: Companion diagnostic indications

TUMOR TYPES	BIOMARKERS	FDA-APPROVED THERAPY
Non-Small Cell Lung Cancer (NSCLC)	*EGFR* exon 19 deletions and *EGFR* exon 21 L858R alterations	Gilotrif® (afatinib), Iressa® (gefitinib), Tagrisso® (osimertinib) or Tarceva® (erlotinib)
	EGFR exon 20 T790M alterations	Tagrisso® (osimertinib)
	ALK rearrangements	Alecensa® (alectinib), Alunbrig® (brigatinib), Xalkori® (crizotinib), or Zykadia® (ceritinib)
	BRAF V600E	Tafinlar® (dabrafenib) in combination with Mekinist® (trametinib)
	MET single nucleotide variants (SNVs) and indels that lead to *MET* exon 14 skipping	Tabrecta® (capmatinib)
Melanoma	*BRAF* V600E	Tafinlar® (dabrafenib) or Zelboraf® (vemurafenib)
	BRAF V600E or V600K	Mekinist® (trametinib) or Cotellic® (cobimetinib), in combination with Zelboraf® (vemurafenib)
Breast Cancer	*ERBB2* (HER2) amplification	Herceptin® (trastuzumab), Kadcyla® (ado-trastuzumab-emtansine), or Perjeta® (pertuzumab)
	PIK3CA C420R, E542K, E545A, E545D [1635G>T only], E545G, E545K, Q546E, Q546R, H1047L, H1047R, and H1047Y alterations	Piqray® (alpelisib)
Colorectal Cancer	*KRAS* wild-type (absence of mutations in codons 12 and 13)	Erbitux® (cetuximab)
	KRAS wild-type (absence of mutations in exons 2, 3 and 4) and *NRAS* wild-type (absence of mutations in exons 2, 3 and 4)	Vectibix® (panitumumab)
Ovarian Cancer	*BRCA1/2* alterations	Lynparza® (olaparib) or Rubraca® (rucaparib)
Cholangiocarcinoma	*FGFR2* fusions and select rearrangements	Pemazyre™ (pemigatinib) or Truseltiq™ (infigratinib)
Prostate Cancer	Homologous Recombination Repair *(HRR) gene (BRCA1, BRCA2, ATM, BARD1, BRIP1, CDK12, CHEK1, CHEK2, FANCL, PALB2, RAD51B, RAD51C, RAD51D and RAD54L)* alterations	Lynparza® (olaparib)
Solid tumors	TMB ≥ 10 mutations per megabase	Keytruda® (pembrolizumab)
	NTRK1/2/3 fusions	Vitrakvi® (larotrectinib)

The test is also used for detection of genomic loss of heterozygosity (LOH) from formalin-fixed, paraffin-embedded (FFPE) ovarian tumor tissue. Positive homologous recombination deficiency (HRD) status (defined as tBRCA-positive and/or LOH high) in ovarian cancer patients is associated with improved progression-free survival (PFS) from Rubraca (rucaparib) maintenance therapy in accordance with the Rubraca product label.

Tarceva® is the registered trademark of OSI Pharmaceuticals, LLC. Zelboraf®, Herceptin®, Perjeta®, Kadcyla®, and Cotellic® are registered trademarks of Genentech, Inc. Gilotrif® is a registered trademark of Boehringer Ingelheim International GmbH. Iressa®, Lynparza®, and Tagrisso® are registered trademarks of the AstraZeneca group of companies. Xalkori® is a registered trademark of Pfizer Inc. Zykadia®, Tafinlar®, Piqray®, Mekinist®, and Tabrecta® are registered trademarks of Novartis AG Corporation Switzerland. Erbitux® is a registered trademark of ImClone LLC, a wholly owned subsidiary of Eli Lilly and Company. Alecensa® is a registered trademark of Chugai Seiyaku Kabushiki Kaisha. Vectibix® is a registered trademark of Immunex Corporation. Rubraca® is a registered trademark of Clovis Oncology, Inc., Pemazyre™ is a registered trademark of Incyte Corporation, Keytruda® is a registered trademark of Merck Sharp & Dohme Corp. Vitrakvi® is a registered trademark of Bayer. Truseltiq™ is a registered trademark of QED Therapeutics, Inc. Alunbrig® is a registered trademark of Takeda Pharmaceutical Company Limited.

Summary of Clinical Studies

Follow-on CDx claims were based on a non-inferiority statistical testing approach using the enrichment design presented in the paper by Li (2016).[1] All studies passed the acceptance criteria specific in each study protocol.

BIOMARKER	POSITIVE PERCENT AGREEMENT (PPA)†	NEGATIVE PERCENT AGREEMENT (NPA)	COMPARATOR METHOD*
EGFR Exon 19 Deletions and L858R	98.1% (106/108)	99.4% (153/154)	cobas® *EGFR* Mutation Test v2
EGFR T790M	98.9% (87/88)	86.1% (93/108)	cobas® *EGFR* Mutation Test v1<br>cobas® *EGFR* Mutation Test v2
ALK Rearrangements	92.9% (78/84)	100% (75/75)	Ventana *ALK* (D5F3) CDx Assay<br>Vysis *ALK* Break-Apart FISH Probe Kit
KRAS	100% (173/173)	100% (154/154)	therascreen® *KRAS* RGQ PCR Kit
ERBB2 (HER2) Amplifications	89.4% (101/113)	98.4% (180/183)	Dako HER2 FISH PharmDx® Kit
BRAF V600	99.4% (166/167)	89.6% (121/135)‡	cobas® *BRAF* V600 Mutation Test
BRAF V600E	99.3% (149/150)	99.2% (121/122)	
BRAF V600 dinucleotide§	96.3% (26/27)	100% (24/24)	THxID® *BRAF* kit

* Cobas® is a trademark of Roche Diagnostics Operations, Inc. Therascreen® is a trademark of Qiagen. PharmDx® is a registered trademark of Dako Denmark A/S. THxID® is a registered trademark of bioMérieux.

† The reference standard used to calculate PPA and NPA is defined as the consensus calls between the two comparator methods – PPA being when FoundationOne CDx and the comparator method(s) identified mutations in mutated patients and NPA being when FoundationOne CDx and the comparator method(s) did not identify mutations in non-mutated patients.

‡ Sensitivity of dinucleotide detection of *BRAF* V600K and V600E was found to be significantly reduced in cobas® test, in particular for samples in which FoundationOne CDx detected the dinucleotides to be of lower than 40% mutant allele frequency (MAF), leading to low NPA values.

§ A study using the THxID® *BRAF* kit (bioMérieux) was conducted with samples with *BRAF* V600 dinucleotide mutation detected by F1CDx and *BRAF* V600 negative samples to provide a better evaluation of V600 dinucleotide concordance.

Current Gene List[2]

Genes with full coding exonic regions included in FoundationOne®CDx for the detection of substitutions, insertion-deletions (indels), and copy-number alterations (CNAs).

ABL1	*ACVR1B*	*AKT1*	*AKT2*	*AKT3*	*ALK*	*ALOX12B*	*AMER1 (FAM123B)*	*APC*
AR	*ARAF*	*ARFRP1*	*ARID1A*	*ASXL1*	*ATM*	*ATR*	*ATRX*	*AURKA*
AURKB	*AXIN1*	*AXL*	*BAP1*	*BARD1*	*BCL2*	*BCL2L1*	*BCL2L2*	*BCL6*
BCOR	*BCORL1*	*BRAF*	*BRCA1*	*BRCA2*	*BRD4*	*BRIP1*	*BTG1*	*BTG2*
BTK	*C11ORF30 (EMSY)*	*CALR*	*CARD11*	*CASP8*	*CBFB*	*CBL*	*CCND1*	*CCND2*
CCND3	*CCNE1*	*CD22*	*CD274 (PD-L1)*	*CD70*	*CD79A*	*CD79B*	*CDC73*	*CDH1*
CDK12	*CDK4*	*CDK6*	*CDK8*	*CDKN1A*	*CDKN1B*	*CDKN2A*	*CDKN2B*	*CDKN2C*
CEBPA	*CHEK1*	*CHEK2*	*CIC*	*CREBBP*	*CRKL*	*CSF1R*	*CSF3R*	*CTCF*
CTNNA1	*CTNNB1*	*CUL3*	*CUL4A*	*CXCR4*	*CYP17A1*	*DAXX*	*DDR1*	*DDR2*
DIS3	*DNMT3A*	*DOT1L*	*EED*	*EGFR*	*EP300*	*EPHA3*	*EPHB1*	*EPHB4*
ERBB2	*ERBB3*	*ERBB4*	*ERCC4*	*ERG*	*ERRFI1*	*ESR1*	*EZH2*	*FAM46C*
FANCA	*FANCC*	*FANCG*	*FANCL*	*FAS*	*FBXW7*	*FGF10*	*FGF12*	*FGF14*
FGF19	*FGF23*	*FGF3*	*FGF4*	*FGF6*	*FGFR1*	*FGFR2*	*FGFR3*	*FGFR4*
FH	*FLCN*	*FLT1*	*FLT3*	*FOXL2*	*FUBP1*	*GABRA6*	*GATA3*	*GATA4*
GATA6	*GID4 (C17ORF39)*	*GNA11*	*GNA13*	*GNAQ*	*GNAS*	*GRM3*	*GSK3B*	*H3F3A*
HDAC1	*HGF*	*HNF1A*	*HRAS*	*HSD3B1*	*ID3*	*IDH1*	*IDH2*	*IGF1R*
IKBKE	*IKZF1*	*INPP4B*	*IRF2*	*IRF4*	*IRS2*	*JAK1*	*JAK2*	*JAK3*
JUN	*KDM5A*	*KDM5C*	*KDM6A*	*KDR*	*KEAP1*	*KEL*	*KIT*	*KLHL6*
KMT2A (MLL)	*KMT2D (MLL2)*	*KRAS*	*LTK*	*LYN*	*MAF*	*MAP2K1 (MEK1)*	*MAP2K2 (MEK2)*	*MAP2K4*
MAP3K1	*MAP3K13*	*MAPK1*	*MCL1*	*MDM2*	*MDM4*	*MED12*	*MEF2B*	*MEN1*
MERTK	*MET*	*MITF*	*MKNK1*	*MLH1*	*MPL*	*MRE11A*	*MSH2*	*MSH3*
MSH6	*MST1R*	*MTAP*	*MTOR*	*MUTYH*	*MYC*	*MYCL (MYCL1)*	*MYCN*	*MYD88*
NBN	*NF1*	*NF2*	*NFE2L2*	*NFKBIA*	*NKX2-1*	*NOTCH1*	*NOTCH2*	*NOTCH3*
NPM1	*NRAS*	*NT5C2*	*NTRK1*	*NTRK2*	*NTRK3*	*P2RY8*	*PALB2*	*PARK2*
PARP1	*PARP2*	*PARP3*	*PAX5*	*PBRM1*	*PDCD1 (PD-1)*	*PDCD1LG2 (PD-L2)*	*PDGFRA*	*PDGFRB*
PDK1	*PIK3C2B*	*PIK3C2G*	*PIK3CA*	*PIK3CB*	*PIK3R1*	*PIM1*	*PMS2*	*POLD1*
POLE	*PPARG*	*PPP2R1A*	*PPP2R2A*	*PRDM1*	*PRKAR1A*	*PRKCI*	*PTCH1*	*PTEN*
PTPN11	*PTPRO*	*QKI*	*RAC1*	*RAD21*	*RAD51*	*RAD51B*	*RAD51C*	*RAD51D*
RAD52	*RAD54L*	*RAF1*	*RARA*	*RB1*	*RBM10*	*REL*	*RET*	*RICTOR*
RNF43	*ROS1*	*RPTOR*	*SDHA*	*SDHB*	*SDHC*	*SDHD*	*SETD2*	*SF3B1*
SGK1	*SMAD2*	*SMAD4*	*SMARCA4*	*SMARCB1*	*SMO*	*SNCAIP*	*SOCS1*	*SOX2*
SOX9	*SPEN*	*SPOP*	*SRC*	*STAG2*	*STAT3*	*STK11*	*SUFU*	*SYK*
TBX3	*TEK*	*TET2*	*TGFBR2*	*TIPARP*	*TNFAIP3*	*TNFRSF14*	*TP53*	*TSC1*
TSC2	*TYRO3*	*U2AF1*	*VEGFA*	*VHL*	*WHSC1 (MMSET)*	*WHSC1L1*	*WT1*	*XPO1*
XRCC2	*ZNF217*	*ZNF703*						

Select Rearrangements[2,3]

Genes with select intronic regions for the detection of gene rearrangements, one gene with a promoter region and one non-coding RNA gene.

ALK	*BCL2*	*BCR*	*BRAF*	*BRCA1*	*BRCA2*	*CD74*	*EGFR*	*ETV4*
ETV5	*ETV6*	*EWSR1*	*EZR*	*FGFR1*	*FGFR2*	*FGFR3*	*KIT*	*KMT2A (MLL)*
MSH2	*MYB*	*MYC*	*NOTCH2*	*NTRK1*	*NTRK2*	*NUTM1*	*PDGFRA*	*RAF1*
RARA	*RET*	*ROS1*	*RSPO2*	*SDC4*	*SLC34A2*	*TERC**	*TERT (PROMOTER ONLY)***	
TMPRSS2								

**TERC* is non-coding RNA gene.
***TERT* is gene with promoter region.

FoundationOne®CDx is a qualitative next-generation sequencing based in vitro diagnostic test for advanced cancer patients with solid tumors and is for prescription use only. The test analyzes 324 genes as well as genomic signatures including microsatellite instability (MSI) and tumor mutational burden (TMB) and is a companion diagnostic to identify patients who may benefit from treatment with specific therapies in accordance with the approved therapeutic product labeling. Additional genomic findings may be reported and are not prescriptive or conclusive for labeled use of any specific therapeutic product. Use of the test does not guarantee a patient will be matched to a treatment. A negative result does not rule out the presence of an alteration. Some patients may require a biopsy. For the complete label, including companion diagnostic indications and important risk information, please visit www.F1CDxLabel.com.

References:

1. Li M. Statistical Methods for Clinical Validation of Follow-On Companion Diagnostic Devices via an External Concordance Study. Statistics in Biopharmaceutical Research 8, 355-363 (2016).
2. Current as of July 2021. Please visit *www.foundationmedicine.com/f1cdx* for the most up-to-date gene list.
3. Refer to our full label for listing of intronic regions at *http://www.F1CDxLabel.com.*

FOUNDATION MEDICINE®

© 2021 Foundation Medicine, Inc. | Foundation Medicine® and FoundationOne® are registered trademarks of Foundation Medicine, Inc.
www.foundationmedicine.com | Tel 888.988.3639 | Fax 617.418.2290 | US-FDX-2000037 V4.0 | RAL-0081
