# Supplementary material for: Precision Oncology in Metastatic Uterine Cancer; Croatian First-Year Experience of the Comprehensive Genomic Profiling in Everyday Clinical Practice
Source: Pathol Oncol Res. 2021 Sep 27;27:1609963. doi: 10.3389/pore.2021.1609963 (PMC8504363; doi:10.3389/pore.2021.1609963)
Supplement: Supplementary file 4 [file DataSheet2.docx]

**Comprenhensive genomic profiling reports for all patients enrolled in the study**

1.

TUMOR TYPE

Unknown primary adenocarcinoma

Genomic Signatures

Microsatellite status - MSI-High

Tumor Mutational Burden - 40 Muts/Mb

Gene Alterations

NRAS G12C

PALB2 L304del

PTEN D107Y, R130G

ARID1A F2141fs*59

KRAS G12D

ATR R1814fs*10

CDK12 Q1291fs*3

CSF3R R755*

JAK1 K860fs*16

MLL2 G5182fs*61

MSH6 F1088fs*5

NOTCH3 G2035fs*50

PBRM1 S652fs*13

TP53 E224fs*1

2.

TUMOR TYPE

Uterus endometrial adenocarcinoma (NOS)

Genomic Signatures

Microsatellite status - MSI-High

Tumor Mutational Burden - 30 Muts/Mb

Gene Alterations

FBXW7 Y545C, R689W

NRAS G12D

PALB2 splice site 211+2T>C

PIK3CA E542V

PTCH1 S1203fs*52

PTEN R130G, V290fs*1

STK11 P281fs*6

ALK A585T

ARID1A D1850fs*33

CCND1 D289del

KEAP1 F478fs*2

KRAS A59T

RNF43 G659fs*41

CBFB P70fs*13

CIC P1116fs*45

CTCF T204fs*26

FLCN H429fs*39

FLT1 R238H

INPP4B L93fs*1

MLL2 G5182fs*61, S102fs*28

NOTCH1 S2486fs*103

SMARCA4 R1005*

WHSC1 (MMSET) E1344fs*91

3.

TUMOR TYPE

Uterus endometrial adenocarcinoma (NOS)

Genomic Signatures

Microsatellite status - MS-Stable

Tumor Mutational Burden - 3 Muts/Mb

Gene Alterations

PTEN N323fs*2, R130G

ARID1A E1780fs*2

PIK3R1 V663fs*1

BCOR N1425S

4.

TUMOR TYPE

Uterus endometrial adenocarcinoma (NOS)

Genomic Signatures

Microsatellite status - MS-Stable

Tumor Mutational Burden - 8 Muts/Mb

Gene Alterations

PIK3R1 I566del

KDM5A amplification

NBN rearrangement exon 6

PPP2R1A P179R

TP53 S241F

5.

TUMOR TYPE

Uterus endometrial adenocarcinoma endometrioid

Genomic Signatures

Microsatellite status - MSI-High

Tumor Mutational Burden - 20 Muts/Mb

Gene Alterations

PIK3CA G118D

ARID1A D1850fs*33

FANCL V167fs*22

ATRX E2265del

HNF1A G292fs*25

MLH1 D584fs*1

MLL2 P2354fs*30, V5311fs*22

MSH3 K383fs*32

RB1 A74fs*4

TEK R522C

TP53 E171fs*3, A138V

6.

TUMOR TYPE

Uterus endometrial adenocarcinoma (NOS)

Genomic Signatures

Microsatellite status - MSI-High

Tumor Mutational Burden - 15 Muts/Mb

Gene Alterations

CTNNB1 S37P

PTCH1 R1308fs*64

PTEN T319fs*1, R130L

ARID1A S1985fs*13

PIK3R1 I405del

CD22 D619N

DNMT3A S708fs*5

7.

TUMOR TYPE

Uterus endometrial adenocarcinoma endometrioid

Genomic Signatures

Microsatellite status - MS-Stable

Tumor Mutational Burden - 6 Muts/Mb

Gene Alterations

BRCA2 duplication exons 25-27

PTEN R233*

ARID1A R2158*, splice site 2732+2T>G

KRAS G13D

CREBBP C1819*

PIK3R1 N453del

U2AF1 S34F

8.

TUMOR TYPE

Uterus endometrial stromal sarcoma

Genomic Signatures

Microsatellite status - MS-Stable

Tumor Mutational Burden - 2 Muts/Mb

Gene Alterations

CDK4 amplification

MDM2 amplification

FRS2 amplification

9.

TUMOR TYPE

Uterus carcinosarcoma

Genomic Signatures

Microsatellite status - MS-Stable

Tumor Mutational Burden - 1 Muts/Mb

Gene Alterations

PIK3CA H1047L

PTEN loss exons 1-8

MDM2 amplification - equivocal†

RAD54L R682*

CCNE1 amplification

DDR1 R514C

TP53 splice site 672+1G>T, I255T

10.

TUMOR TYPE

Uterus adenosarcoma

Genomic Signatures

Microsatellite status - MS-Stable

Tumor Mutational Burden - 2 Muts/Mb

Gene Alterations

No reportable genomic alterations were detected. See below for more

information.

11.

TUMOR TYPE

Uterus endometrial adenocarcinoma endometrioid

Genomic Signatures

Microsatellite status - MSI-High

Tumor Mutational Burden - 21 Muts/Mb

Gene Alterations

PIK3CA R88Q

ARID1A P1878fs*5, E2250fs*27

ATR V2620fs*35

BCORL1 P1681fs*20

CD79A R131fs*61

DNMT3A A157fs*30

MLL2 R5027*

MRE11A F399fs*29

MSH3 K383fs*32

PBRM1 I873fs*2

12.

TUMOR TYPE

Uterus endometrial adenocarcinoma (NOS)

Genomic Signatures

Microsatellite status – Cannot Be Determined

Tumor Mutational Burden - Cannot Be Determined

Gene Alterations

PIKRCA E545K

KRAS G12A

BRD4 Q1017fs*50

CASP8 R250Q

TP53 G245S

U2AF1 S34F

13.

TUMOR TYPE

Uterus endometrial adenocarcinoma endometrioid

Genomic Signatures

Microsatellite status – MS-Stable

Tumor Mutational Burden - 0 Muts/Mb

Gene Alterations

PIK3CA E542K

CIC D464fs*8

TP53 R175H

14.

TUMOR TYPE

Uterus endometrial adenocarcinoma (NOS)

Genomic Signatures

Microsatellite status - MS-Stable

Tumor Mutational Burden - 0 Muts/Mb

Gene Alterations

AKT1 E17K

CTNNB1 S45F

ARID1A S2126fs*24

PTPRO F265fs*8

15.

TUMOR TYPE

Uterus endometrial adenocarcinoma (NOS)

Genomic Signatures

Microsatellite status - MS-Stable

Tumor Mutational Burden - 4 Muts/Mb

Gene Alterations

CTNNB1 S37A

PIK3CA E542K

ARID1A M362fs*38, K622*

CBFB E84*

NFKBIA L303fs*17

16.

TUMOR TYPE

Uterus endometrial adenocarcinoma endometrioid

Genomic Signatures

Microsatellite status - MSI-High

Tumor Mutational Burden - 13 Muts/Mb

Gene Alterations

CTNNB1 G34E

NF2 splice site 517-2A>G

PTEN Y336*, N323fs*2

ARID1A H1524fs*7, F2141fs*59

PIK3R1 splice site 1746-2A>C

BCOR N1425S

EP300 S12*

EZH2 R207* - subclonal†

JAK1 P430fs*2

MLL2 Q3839fs*173

17.

TUMOR TYPE

Ovary endometrioid adenocarcinoma

Genomic Signatures

Loss of Heterozygosity score - 6.0%

Microsatellite status -MS-Stable

Tumor Mutational Burden - 9 Muts/Mb

Gene Alterations

For a complete list of the genes assayed, please refer to the Appendix.

PALB2 Y1183*

KRAS G12D

2 Disease relevant genes with no reportable alterations: BRCA1, BRCA2

18.

TUMOR TYPE

Uterus endometrial adenocarcinoma endometrioid

Genomic Signatures

Microsatellite status -MSI-High

Tumor Mutational Burden - 32 Muts/Mb

Gene Alterations

ATMG696*

BRCA2 E2981fs*37

PIK3CA R88Q, Q546K

ARID1A F2141fs*59

MET D1117G - subclonal†

RNF43 G659fs*41

BCORL1 P1681fs*20

CIC splice site 4460-2A>G,

A652fs*76

CREBBP L524fs*6

CTCF Y64fs*16

EP300 K1047fs*12

FANCC Q17*

JAK1 P430fs*2

MSH3 K383fs*32

MSH6 F1088fs*2

PBRM1 R710*

SMARCA4 R1243W - subclonal†

WT1 G269D

19.

TUMOR TYPE

Uterus endometrial adenocarcinoma (NOS)

Genomic Signatures

Microsatellite status -MS-Stable

Tumor Mutational Burden - 1 Muts/Mb

Gene Alterations

AKT1 E17K

MTORM2327I

PIK3R1 N625fs*7

TP53 A276G

20.

TUMOR TYPE

Uterus endometrial adenocarcinoma endometrioid

Genomic Signatures

Tumor Mutational Burden - 11 Muts/Mb

Microsatellite status -MS-Stable

Gene Alterations

FGFR2 S252W

NF2 Y153fs*20

PALB2 V132fs*45

PIK3CA P471L

PTEN L318fs*23 - subclonal, G132V

KDM6A T640fs*50 – subclonal

21.

TUMOR TYPE

Uterus endometrial adenocarcinoma (NOS)

Genomic Signatures

Microsatellite status -MSI-High

Tumor Mutational Burden - 18 Muts/Mb

Gene Alterations

PTEN D92H

ARID1A Q1519fs*8

PIK3CB E1051K

CASP8 R194fs*17

NOTCH3 G1318fs*245

SOX9 P346fs*37

TP53 V218M

22.

TUMOR TYPE

Uterus endometrial adenocarcinoma papillary serous

Genomic Signatures

Microsatellite status -MS-Stable

Tumor Mutational Burden - 4 Muts/Mb

Gene Alterations

PIK3R1 E451_Y452del

CDC73 R229fs*27

PPP2R1A S256F

TP53 R248P

23.

TUMOR TYPE

Uterus endometrial adenocarcinoma papillary serous

Genomic Signatures

Microsatellite status -MS-Stable

Tumor Mutational Burden - 1 Muts/Mb

Gene Alterations

PIK3CA E545K

ARID1A Q586*

ERBB3 G582W

ALOX12B R548W

CSF1R inversion exons 2-13

RPTOR amplification

TP53 C176W

24.

TUMOR TYPE

Uterus leiomyosarcoma

Genomic Signatures

Microsatellite status -MS-Stable

Tumor Mutational Burden - 5 Muts/Mb

Gene Alterations

CIC loss

25.

TUMOR TYPE

Uterus endometrial adenocarcinoma (NOS)

Genomic Signatures

Microsatellite status -MS-Stable

Tumor Mutational Burden - 3 Muts/Mb

Gene Alterations

NRAS G13R

PTEN T78fs*5

ARID1A Q2026*

PIK3R1 L570_I571insN

BCOR N1425S

NFE2L2 E79G

SOX9 D12fs*49

26.

TUMOR TYPE

Uterus endometrial adenocarcinoma endometrioid

Genomic Signatures

Microsatellite status -MSI-High

Tumor Mutational Burden - 28 Muts/Mb

Gene Alterations

BRCA2 N1784fs*7

PIK3CA R88Q - subclonal, R93Q, C378R†

PTEN R130G

ARID1A P947fs*21

ERBB3 A245V

RNF43 G659fs*41

ESR1 L536R

FUBP1 S11fs*43

MAP2K4 A279T - subclonal†

NOTCH3 splice site 3719-1G>T

PTPN11 T468M - subclonal†

TP53 T125M

27.

TUMOR TYPE

Uterus endometrial adenocarcinoma clear cell

Genomic Signatures

Tumor Mutational Burden - 25 Muts/Mb

Microsatellite status -MS-Stable

Gene Alterations

ERBB2 V842I

FBXW7 R465H

NF1 R1276*, Q1835*

ARID1A R1276*

CDH1 rearrangement exon 15

ERRFI1 R244*

GNAS R201H

KDM5C R778*

MSH6 Q1328*, F1088fs*2

RB1 R661W - subclonal†

SMARCA4 T910M

TP53 R273C, R181C

28.

TUMOR TYPE

Uterus leiomyosarcoma

Genomic Signatures

Microsatellite status -MS-Stable

Tumor Mutational Burden - 3 Muts/Mb

Gene Alterations

ATRX- T2228fs*18

CDKN1B-R154fs*69

RB1-rearr.ex2

TP53- G266R

29.

TUMOR TYPE

Uterus leiomyosarcoma

Genomic Signatures

Microsatellite status -MS-Stable

Tumor Mutational Burden - 2 Muts/Mb

Gene Alterations

ARID1A loss

EPHA3 R750L

TP53 splice site 86_96+4delACAACGTTCTGGTAA

30.

TUMOR TYPE

Ovary endometrioid adenocarcinoma

Genomic Signatures

Loss of Heterozygosity score - 3.8%

Microsatellite status -MS-Stable

Tumor Mutational Burden - 3 Muts/Mb

Gene Alterations

AKT1 E17K

CTNNB1 S33C

FGFR2 S252W - subclonal†

PIK3CA H1048R

BCOR N1425S

2 Disease relevant genes with no reportable alterations: BRCA1, BRCA2

31.

TUMOR TYPE

Uterus carcinosarcoma

Genomic Signatures

Microsatellite status -MS-Stable

Tumor Mutational Burden - 2 Muts/Mb

Gene Alterations

PIK3CA T1025S

TP53 R342*

32.

TUMOR TYPE

Uterus endometrial adenocarcinoma papillary serous

Genomic Signatures

Microsatellite status -MS-Stable

Tumor Mutational Burden - 3 Muts/Mb

Gene Alterations

PIK3CA E542A

PTEN Q171*, C83fs*16

FGFR1 V561M

PPP2R1A S256F

TP53 K132M
